# Cardiovascular Risk Factor Profiles for Peripheral Artery Disease and Carotid Atherosclerosis among Chinese Older People: A Population-Based Study

**DOI:** 10.1371/journal.pone.0085927

**Published:** 2014-01-17

**Authors:** Yajun Liang, Zhongrui Yan, Binglun Sun, Chuanzhu Cai, Hui Jiang, Aiqin Song, Chengxuan Qiu

**Affiliations:** 1 School of Public Health, Jining Medical University, Shandong, P. R. China; 2 Aging Research Center, Department of Neurobiology, Care Sciences and Society, Karolinska Institutet-Stockholm University, Stockholm, Sweden; 3 Department of Neurology, Jining First People’s Hospital, Shandong, P. R. China; 4 Xing Long Zhuang Coal Mine Hospital, Yankuang Group, Shandong, P. R. China; University of Kansas Medical Center, United States of America

## Abstract

**Objectives:**

Epidemiological data concerning atherosclerotic disease among older people in rural China are sparse. We seek to determine prevalence and cardiovascular risk factor profiles for peripheral artery disease (PAD) and carotid atherosclerosis (CAS) among Chinese older people living in a rural community.

**Methods:**

This cross-sectional study included 1499 participants (age ≥60 years, 59.0% women) of the Confucius Hometown Aging Project in Shandong, China. From June 2010–July 2011, data were collected through interviews, clinical examinations, and laboratory tests. PAD was defined as an ankle-brachial index ≤0.9. Carotid intima-media thickness (cIMT) and carotid artery stenosis were assessed by ultrasonography. We defined moderate stenosis as carotid stenosis ≥50%, and severe stenosis as carotid stenosis ≥70%. cIMT≥1.81 mm was considered as an increased cIMT (a measure of CAS). Data were analyzed with multiple logistic models.

**Results:**

The prevalence was 5.7% for PAD, 8.9% for moderate stenosis, 1.8% for severe stenosis, and 11.2% for increased cIMT. After controlling for multiple potential confounders, diabetes, an increased low-density lipoprotein cholesterol (LDL-C)/high-density lipoprotein cholesterol (HDL-C) ratio, and hypertension were significantly or marginally associated with PAD. Ever smoking, hypertension, and an increased LDL-C/HDL-C ratio were significantly associated with an increased likelihood of increased cIMT. An increasing number of those cardiovascular risk factors were significantly associated with an increasing odds ratio of PAD and increased cIMT, respectively (p for linear trend <0.001).

**Conclusion:**

Among Chinese older people living in a rural community, PAD, carotid artery stenosis, and an increased cIMT are relatively uncommon. Cardiovascular risk factor profiles for PAD and CAS are slightly different, with hypertension and an increased LDL-C/HDL-C ratio being associated with an increased likelihood of both PAD and increased cIMT.

## Introduction

The prevalence of clinical and subclinical atherosclerotic disorders increases with advancing age [1], [2]. Besides age, the atherosclerotic process can be accelerated by cardiovascular risk factors such as smoking, hypertension, high cholesterol, and diabetes [3]–[5]. Population-based studies have suggested that subclinical atherosclerotic diseases such as peripheral artery disease (PAD) and carotid atherosclerosis (CAS) (e.g., increased carotid intima-media thickness [cIMT], carotid artery stenosis, and irregular carotid plaque) are associated with an increased risk of not only cardiovascular disease [6]–[8], but also cognitive and physical impairment [9]–[11]. Therefore, assessing subclinical atherosclerosis among older people may help to identify a subgroup at high risk for subsequent development of cardiovascular events and dementing disorders that can be targeted for early preventative and therapeutic intervention.

The presence and severity of atherosclerosis can be assessed by using noninvasive techniques. A low ankle-brachial index (ABI) is considered as a surrogate marker for generalized PAD, while an increased cIMT is an intermediate phenotype for early central artery atherosclerosis. Both a low ABI and an increased cIMT, as markers of systemic atherosclerotic disease, are highly correlated [12]. However, few studies have investigated whether PAD and CAS have different cardiovascular risk factor profiles among older adults.

Previously, population-based studies have shown that individual cardiovascular risk factors, such as smoking and high blood pressure, are associated with PAD and CAS among Chinese older people [13]–[15]. We hypothesize that an aggregation of multiple cardiovascular risk factors may be more strongly associated with measures of atherosclerosis than individual factors in older people, where PAD and CAS may have different cardiovascular risk factor profiles. In this population-based study, we seek (1) to determine the prevalence of PAD, carotid artery stenosis, and increased cIMT among Chinese older people living in a rural community, and (2) to explore whether the cardiovascular risk factor profiles for PAD and CAS (measured as increased cIMT) are different.

## Methods

### Ethics Statement

The protocols, instruments, and the consent procedure of the Confucius Hometown Aging Project (CHAP) were approved by the Ethics Committee at Jining First People’s Hospital of Jining Medical University in Shandong, China. Written informed consent was obtained from participants, or from informants, in the case of cognitive impairment. All eligible participants who declined to participate were not disadvantaged in any way by not participating in the study. Research within CHAP had been conducted according to the principles expressed in the Declaration of Helsinki.

### Study Population

The study population included participants of the CHAP as previously reported [16]. Eligible subjects of CHAP included all people aged 60 years or over who were registered in the Xing Long Zhuang community in June 2010. The community was located approximately 20 km away from Qufu (Hometown of Confucius), Shandong, China. The CHAP was conducted by Jining First People’s Hospital and Jining Medical University in Shandong, China, in collaboration with the Aging Research Center at Karolinska Institutet-Stockholm University, Stockholm, Sweden. The overall aim of CHAP was to investigate health in aging by focusing on the possible roles of cardiovascular risk factors and atherosclerosis in the aging process.

Baseline examination was conducted from June 2010–July 2011, during which extensive data were collected through a face-to-face interview, a clinical examination, and laboratory tests. All assessments were carried out by nurses, physicians, and laboratory technicians from Xing Long Zhuang Coal Mine Hospital that provides health care services to residents in the local community. Before the examination, the research staff for CHAP was trained by specialists and senior researchers from the Aging Research Center at Karolinska Institutet-Stockholm University, Stockholm, Sweden. Of all eligible subjects (n = 1743), 204 refused to participate or moved out of the area, and 40 had missing data on either laboratory tests or measures of atherosclerosis, leaving 1499 subjects (86.0%) for the current analysis.

### Data Collection and Definitions

Epidemiological data were collected following a questionnaire that was developed from the World Health Organization STEPwise approach to Surveillance [17] and the Study on Global Ageing and Adult Health [18]. We collected data on age, sex, education, lifestyle factors (e.g., smoking, alcohol consumption, and physical activity at leisure time), medical history (e.g., hypertension, diabetes, and cardiovascular diseases), diets (e.g., frequency of meat consumption), and use of medications (e.g., antihypertensive agents, blood glucose-lowering drugs, and cholesterol-lowering medication) two weeks prior to the interview. Smoking status was dichotomized as never vs. ever (current or former) smoking. Alcohol consumption was assessed based on the frequency and amount of alcohol intake in a typical drinking day, and was dichotomized as yes vs. no. Physical inactivity was defined as less than 600 metabolic equivalent-minutes of leisure-time physical activity per week [16]. Frequency of meat consumption (e.g., pork, beef, and lamb) was categorized into <1 time/week, 1–5 times/week, and almost daily consumption.

Weight and height were measured with participants wearing light clothes and no shoes. Body mass index (BMI) was calculated as weight in kilograms divided by height in meters squared. Overweight was defined as BMI 25–29.9 kg/m^2^, and obesity as BMI ≥30 kg/m^2^.

After at least a five-min rest, arterial blood pressure (Korotkoff systolic phase I and diastolic phase V) was measured in the sitting position on the right arm using a mercury sphygmomanometer with the cuff maintained at heart level [19]. Blood pressure was measured twice at a one-min interval, and the mean of two readings was used in the analysis. Hypertension was defined as a blood pressure ≥140/90 mm Hg or currently using antihypertensive drugs; stage 1 hypertension was defined as a blood pressure of 140–159/90–99 mm Hg without using antihypertensive agents, and stage 2 hypertension was defined as blood pressure ≥160/100 mm Hg or currently using antihypertensive drugs [19].

Systolic pressures at ankle and brachial arteries were measured using a Doppler ultrasonic device (8-MHz, Model L500VA, Vista AVS™, USA) following a standard method [20]. ABI for each side was calculated by dividing ankle systolic pressure on one side by the higher of brachial systolic pressure measurements on both sides. The lower ABI of both sides was used in the analysis. PAD was defined as ABI ≤0.90 [20].

A clinical sonographer assessed cIMT and carotid artery stenosis on the right and left internal carotid artery with color Doppler ultrasonography (Vivid 7 ultrasound system and a 7- to 10-MHz transducer) by following a standardized protocol [21]. We measured cIMT three times at one visit, and recorded the readings to the nearest 0.1 mm. The mean of three measurements was used in the analysis. Moderate carotid artery stenosis was defined as a stenosis of 50% or greater, and severe carotid artery stenosis as a stenosis of 70% or greater [22]. Increased cIMT was defined as cIMT ≥1.81 mm on either side [23].

After overnight fasting, a peripheral blood sample was taken at the hospital during the visit. Fasting plasma glucose (FPG), total cholesterol, triglycerides, high-density lipoprotein cholesterol (HDL-C), low-density lipoprotein cholesterol (LDL-C), and lipoprotein (a) [Lp(a)] were measured using an automatic Biochemical Analyzer (Olympus AU400, Olympus Optical Co., Ltd, Tokyo, Japan) at the hospital laboratory [16]. Diabetes was defined as a FPG ≥7.0 mmol/L or currently using blood glucose-lowering medications or insulin injections, while prediabetes was defined as a FPG of 6.0–6.99 mmol/L in participants free of diabetes [24]. High total cholesterol was defined as serum total cholesterol level >6.2 mmol/L or receiving cholesterol-lowering medications; high triglycerides was considered as ≥2.3 mmol/L or receiving cholesterol-lowering medications; low HDL-C was characterized as <1.0 mmol/L in men or <1.3 mmol/L in women or currently receiving cholesterol-lowering medications; and high LDL-C was defined as ≥4.1 mmol/L or receiving cholesterol-lowering medications [25]. The LDL-C/HDL-C ratio was categorized into tertiles. A serum Lp(a) ≥0.5 g/L was considered as high Lp(a) [26].

### Statistical Analysis

The characteristics of study participants by sex were compared using a *t*-test for continuous variables with a normal distribution or a *chi*-square test for categorical variables. Logistic regression analyses were performed to estimate odds ratios and 95% confidence intervals of PAD and increased cIMT associated with individual and clustering of cardiovascular risk factors. The cardiovascular risk factor profile (clustering) was assessed by counting the number of cardiovascular risk factors that were potentially associated with an increased odds ratio of PAD and increased cIMT, respectively. We reported results from two models: model 1 was controlled for age and sex, and model 2 was further controlled for all examined cardiovascular risk factors. IBM SPSS Statistics 19 for Windows (IBM SPSS Inc., Chicago, Illinois, U.S.) was used for all analyses.

## Results

The mean age of all participants was 68.5 years (SD, 4.9), and 59.0% were women. Men were more likely than women to smoke, drink alcohol, participate in physical activity, and consume meats (p<0.001) (Table 1). In addition, compared to men, women had higher levels of systolic pressure, FPG, total cholesterol, triglycerides, HDL-C, LDL-C, LDL-C/HDL-C ratio, and Lp(a) (p≤0.01), but a lower level of ABI (p = 0.001). There was no significant sex difference regarding average age, BMI, and diastolic pressure (p>0.05).

**Table 1:** Characteristics of study participants by sex.

Characteristics*	Total (n = 1499)	Men (n = 615)	Women (n = 884)	p-value†
Age (years), mean (SD)	68.5 (4.9)	68.7 (5.0)	68.4 (4.9)	0.42
Ever smoking, n (%)	451 (30.1)	367 (59.7)	84 (9.5)	<0.001
Alcohol intake, n (%)	278 (18.7)	235 (38.4)	43 (4.9)	<0.001
Physical inactivity, n (%)	1254 (83.7)	464 (75.4)	790 (89.4)	<0.001
Frequency of meat consumption, n (%)				
<1 time/week	619 (41.3)	214 (34.8)	405 (45.8)	
1–5 times/week	567 (37.8)	248 (40.3)	319 (36.1)	
Almost daily	313 (20.9)	153 (24.9)	160 (18.1)	<0.001
Body mass index (kg/m^2^), mean (SD)	26.3 (3.8)	26.1 (3.9)	26.5 (3.8)	0.07
Systolic pressure (mm Hg), mean (SD)	148.9 (23.0)	145.2 (21.9)	151.4 (23.4)	<0.001
Diastolic pressure (mm Hg), mean (SD)	87.5 (12.2)	88.1 (11.8)	87.0 (12.4)	0.09
FPG (mmol/L), mean (SD)	5.7 (1.6)	5.5 (1.5)	5.8 (1.6)	0.01
Total cholesterol (mmol/L), mean (SD)	5.3 (1.0)	4.9 (0.9)	5.6 (1.0)	<0.001
Triglycerides (mmol/L), median (IQR)	1.3 (1.0–1.9)	1.2 (0.8–1.6)	1.5 (1.1–2.0)	<0.001
HDL-C (mmol/L), mean (SD)	1.4 (0.3)	1.3 (0.3)	1.4 (0.3)	<0.001
LDL-C (mmol/L), mean (SD)	2.9 (0.7)	2.7 (0.6)	3.1 (0.7)	<0.001
LDL-C/HDL-C ratio, mean (SD)	2.2 (0.6)	2.1 (0.7)	2.2 (0.6)	0.003
Lipoprotein(a) (g/L), median (IQR)	1.2 (0.6–2.7)	1.0 (0.5–2.3)	1.3 (0.6–3.1)	<0.001
Ankle-brachial index, mean (SD)	1.07 (0.11)	1.08 (0.11)	1.06 (0.11)	0.001
Peripheral artery disease, n (%)	85 (5.7)	32 (5.2)	53 (6.0)	0.51
Carotid artery stenosis				
Moderate stenosis (≥50%), n (%)	121 (8.9)	71 (12.7)	50 (6.2)	<0.001
Severe stenosis (≥70%), n (%)	24 (1.8)	11 (2.0)	13 (1.6)	0.64
Increased cIMT, n (%)	168 (11.2)	97 (15.8)	71 (8.0)	<0.001

Abbreviations: SD: standard deviation; FPG: fasting plasma glucose; IQR: interquartile range; HDL-C: high-density lipoprotein cholesterol; LDL-C: low-density lipoprotein cholesterol; cIMT: carotid intima-media thickness.

Numbers of subjects with missing values were 10 for alcohol consumption, 19 for body mass index, and 138 for carotid artery stenosis.

p-value is for the test of difference between men and women.

The overall prevalence was 5.7% for PAD and 11.2% for an increased cIMT. Of the 1361 (90.8%) persons with data on carotid artery stenosis, the prevalence was 8.9% for moderate carotid artery stenosis (stenosis ≥50%) and 1.8% for severe carotid artery stenosis (stenosis ≥70%). Compared to women, men had a higher prevalence of moderate carotid stenosis (men vs. women: 12.7% vs. 6.2%, p<0.001) and an increased cIMT (15.8% vs. 8.0%, p<0.001). There was no significant sex difference in the prevalence of PAD (5.2% vs. 6.0%, p = 0.51) and severe carotid artery stenosis (2.0% vs. 1.6%, p = 0.64) (Table 1).

When controlling for age and sex, PAD was significantly associated with hypertension (stage 2), diabetes, high cholesterol, high LDL-C, and an increased LDL-C/HDL-C ratio, whereas ever smoking, less frequent meat consumption, hypertension, diabetes, high cholesterol, high LDL-C, and an increased LDL-C/HDL-C ratio were significantly associated with an increased odds ratio of increased cIMT (Table 2, model 1). In the multi-adjusted logistic model (Table 2, model 2), PAD was significantly or marginally associated with hypertension, diabetes, and a high LDL-C/HDL-C ratio; and ever smoking, less frequent meat consumption, hypertension, and an increased LDL-C/HDL-C ratio were significantly associated with increased cIMT. None of alcohol consumption, physical inactivity, overweight, obesity, high triglycerides, low HDL-C, and high Lp(a) was significantly associated with either PAD or increased cIMT.

**Table 2:** Association of cardiovascular risk factors with peripheral artery disease and increased carotid intima-media thickness.

Cardiovascular risk factors	Peripheral artery disease			Increased carotid intima-media thickness		
	No. of cases	Odds ratio (95% confidence interval)		No. of cases	Odds ratio (95% confidence interval)	
		Model 1*	Model 2†		Model 1*	Model 2†
Smoking status						
Never	58	1.00 (reference)	1.00 (reference)	93	1.00 (reference)	1.00 (reference)
Ever	27	1.26 (0.73–2.20)	1.27 (0.71–2.27)	75	1.51 (1.03–2.23)	1.54 (1.03–2.31)
Alcohol intake						
No	71	1.00 (reference)	1.00 (reference)	131	1.00 (reference)	1.00 (reference)
Yes	14	1.03 (0.54–1.99)	1.24 (0.62–2.49)	36	0.87 (0.56–1.33)	0.89 (0.56–1.42)
Physical inactivity						
No	11	1.00 (reference)	1.00 (reference)	28	1.00 (reference)	1.00 (reference)
Yes	74	1.12 (0.57–2.18)	0.93 (0.47–1.86)	140	1.10 (0.71–1.72)	0.99 (0.62–1.59)
Meat consumption						
<1 time/week	32	1.00 (reference)	1.00 (reference)	73	1.00 (reference)	1.00 (reference)
1–5 times/week	33	1.20 (0.72–1.99)	1.14 (0.68–1.91)	69	0.99 (0.69–1.41)	0.94 (0.65–1.36)
Almost daily	20	1.34 (0.75–2.42)	1.26 (0.69–2.31)	26	0.61 (0.38–0.99)	0.60 (0.37–0.98)
BMI (kg/m^2^) status						
Normal	36	1.00 (reference)	1.00 (reference)	62	1.00 (reference)	1.00 (reference)
Overweight	38	0.95 (0.59–1.53)	0.72 (0.44–1.19)	86	1.18 (0.83–1.67)	0.95 (0.66–1.37)
Obesity	10	0.95 (0.46–1.97)	0.66 (0.31–1.40)	17	0.96 (0.54–1.69)	0.70 (0.39–1.28)
Hypertension						
No	12	1.00 (reference)	1.00 (reference)	22	1.00 (reference)	1.00 (reference)
Stage 1	8	0.94 (0.38–2.34)	0.99 (0.39–2.52)	21	1.44 (0.77–2.70)	1.38 (0.73–2.61)
Stage 2	65	1.99 (1.06–3.75)	1.91 (0.97–3.74)	125	2.45 (1.52–3.95)	2.21 (1.35–3.65)
Diabetic status						
No	35	1.00 (reference)	1.00 (reference)	90	1.00 (reference)	1.00 (reference)
Prediabetes	14	1.70 (0.89–3.24)	1.58 (0.82–3.06)	21	1.00 (0.61–1.66)	0.78 (0.46–1.33)
Diabetes	36	2.46 (1.51–4.00)	2.21 (1.32–3.69)	57	1.50 (1.05–2.15)	1.26 (0.86–1.83)
High cholesterol						
No	49	1.00 (reference)	1.00 (reference)	106	1.00 (reference)	1.00 (reference)
Yes	36	1.57 (1.00–2.46)	1.64 (0.93–2.90)	62	1.39 (0.99–1.96)	1.30 (0.83–2.03)
High triglycerides						
No	59	1.00 (reference)	1.00 (reference)	114	1.00 (reference)	1.00 (reference)
Yes	26	1.18 (0.73–1.91)	0.68 (0.36–1.26)	54	1.33 (0.94–1.89)	0.93 (0.58–1.49)
Low HDL-C						
No	45	1.00 (reference)	1.00 (reference)	99	1.00 (reference)	1.00 (reference)
Yes	40	1.30 (0.83–2.04)	1.00 (0.58–1.71)	69	1.21 (0.86–1.69)	0.96 (0.63–1.46)
High LDL-C						
No	59	1.00 (reference)	1.00 (reference)	122	1.00 (reference)	1.00 (reference)
Yes	26	1.64 (1.01–2.66)	1.32 (0.73–2.37)	46	1.47 (1.01–2.12)	1.25 (0.79–1.99)
LDL-C/HDL-C ratio (tertile)						
Lower (<1.89)	15	1.00 (reference)	1.00 (reference)	36	1.00 (reference)	1.00 (reference)
Middle (1.89–2.43)	29	2.03 (1.07–3.86)	1.89 (0.99–3.63)	59	1.74 (1.12–2.70)	1.66 (1.06–2.60)
Upper (≥2.44)	41	2.87 (1.56–5.29)	2.56 (1.37–4.81)	73	2.35 (1.53–3.59)	2.06 (1.32–3.21)
High lipoprotein(a)						
No	15	1.00 (reference)	1.00 (reference)	30	1.00 (reference)	1.00 (reference)
Yes	69	1.19 (0.67–2.12)	1.28 (0.70–2.34)	138	1.31 (0.86–1.99)	1.29 (0.83–1.99)

Abbreviations: BMI: body mass index; HDL-C: high-density lipoprotein cholesterol; LDL-C: low-density lipoprotein cholesterol.

Controlled for age and sex.

Odds ratio and 95% confidence interval were derived from the model that included age, sex, smoking status, alcohol intake, physical inactivity, meat consumption, overweight or obesity, hypertension, diabetes or prediabetes, and one measure of high cholesterol, high triglycerides, low HDL-C, high LDL-C, LDL-C/HDL-C ratio, and high lipoprotein(a).

When stage 2 hypertension, diabetes, and an increased LDL-C/HDL-C ratio (middle or upper tertile) were aggregated, an increasing burden of cardiovascular risk factors was significantly associated with an increased likelihood of PAD (p for linear trend <0.001) (Table 3). The linear trend of association remained statistically significant when multiple variables were added to the model (Table 3, model 2). Similarly, when ever smoking, hypertension, and an increased LDL-C/HDL-C ratio (middle or upper tertile) were aggregated, an increasing number of these cardiovascular risk factors were significantly associated with an increased odds ratio of increased cIMT, even in the multivariable model (p for linear trend <0.001) (Table 3, models 1 and 2).

**Table 3:** Association of peripheral artery disease and increased carotid intima-media thickness with a clustering of cardiovascular risk factors.

No. ofcardiovascularrisk factors*	Peripheral artery disease				Increased carotid intima-media thickness			
	No. ofsubjects	No. ofcases	Odds ratio (95% confidence interval)		No. ofsubjects	No. ofcases	Odds ratio (95% confidence interval)	
			Model 1†	Model 2‡			Model 1†	Model 2§
0	370	8	1.00 (reference)	1.00 (reference)	295	18	1.00 (reference)	1.00 (reference)
1	590	28	2.02 (0.91–4.51)	2.51 (1.07–5.87)	654	52	1.20 (0.68–2.09)	1.19 (0.68–2.09)
2	411	33	3.66 (1.66–8.07)	4.84 (2.07–11.30)	453	73	2.48 (1.43–4.28)	2.42 (1.38–4.23)
3	128	16	5.75 (2.37–13.92)	7.61 (2.95–19.66)	97	25	4.02 (2.04–7.91)	3.64 (1.80–7.36)
p for linear trend			<0.001	<0.001			<0.001	<0.001

The cardiovascular risk factor profile consisted of stage 2 hypertension, diabetes, and an increased LDL-C/HDL-C ratio (middle or upper tertile vs. lower tertile) for peripheral artery disease, and ever smoking, stage 2 hypertension, and an increased LDL-C/HDL-C ratio for increased carotid intima-media thickness.

Controlled for age and sex.

Controlled for age, sex, ever smoking, alcohol intake, physical inactivity, meat consumption, and overweight or obesity.

Controlled for age, sex, alcohol intake, physical inactivity, meat consumption, overweight or obesity, and diabetes.

## Discussion

In this population-based study of Chinese older people, we found that the prevalence was 5.7% for PAD, 8.9% for moderate carotid artery stenosis, 1.8% for severe stenosis, and 11.2% for increased cIMT. Hypertension, diabetes, and an increased LDL-C/HDL-C ratio were associated with an increased odds ratio of PAD, likewise ever smoking, less frequent meat consumption, hypertension, and an increased LDL-C/HDL-C ratio were associated with an increased likelihood of increased cIMT. Furthermore, an aggregation of multiple cardiovascular risk factors was strongly associated with an increased likelihood of PAD and increased cIMT, where the cardiovascular risk factor profiles for PAD and increased cIMT were slightly different.

The prevalence of PAD in our population was generally comparable to the reports from a study of elders living in the rural communities in Beijing [15], yet lower compared to those living in the urban areas [13]. The differences in lifestyle factors and socioeconomic status of study participants might partly explain the variations in prevalence between urban and rural residents. We found no sex difference in the prevalence of PAD, which was consistent with findings from previous studies [15]. In addition, our study showed that the overall and the sex-specific prevalence rates of moderate and severe carotid artery stenosis were in good agreement with the data derived from systematic reviews and meta-analyses [22], [27]. Men had a higher prevalence of moderate carotid stenosis and increased cIMT than women, in which the gender difference may be partly due to the anti-inflammatory effect of sex hormones (e.g., estrogen) in the atherogenic process [28].

Earlier population-based studies showed that conventional cardiovascular risk factors such as smoking, hypertension, and diabetes were associated with PAD and CAS in young, middle-aged, and older adults [4], [29]. In line with these studies, we found that diabetes was associated with PAD, but the association with increased cIMT was partly accounted by other cardiovascular risk factors. Smoking was associated with an increased cIMT, possibly by inducing a pro-inflammatory remodeling phenotype in small muscle cell [30]. We found no association of alcohol intake, physical inactivity, and overweight or obesity with either PAD or increased cIMT among Chinese older people. The finding of an association of almost daily meat consumption with a decreased likelihood of an enlarged cIMT, but not with PAD, deserves further investigation by using quantitative measures of meat consumption while also taking into account various types of meat (e.g., red vs. white meat).

The LDL-C/HDL-C ratio has been increasingly recognized as a stronger predictor of cardiovascular events than total cholesterol and its components (e.g., LDL-C and HDL-C) [5]. Preceding research has suggested that an increased LDL-C/HDL-C ratio not only predicts severe atherosclerosis, but also plays a role in the progression of cIMT [31], [32]. The associations of the LDL-C/HDL-C ratio with PAD and CAS have been rarely investigated among older people. One of the main findings of this study was that, of all the examined individual cardiovascular risk factors, the increased LDL-C/HDL-C ratio had the strongest association with both PAD and CAS measured as increased cIMT.

The elevated serum Lp(a) has been associated with an increased risk for cardiovascular disease among older people [33]. Lp(a) may also be a risk factor for increased cIMT and coronary atherosclerosis, even among individuals without clinical cardiovascular disease [34]. Studies from European populations suggest that Lp(a) is independently associated with PAD among middle-aged and older people [35]. However, we did not find an independent association of high Lp(a) with either PAD or increased cIMT in Chinese older people. It is unclear, to what extent, differences in genetic background, characteristics of the study sample, and environmental factors (e.g., diets and lifestyles) between Asian and Western populations may explain the different findings.

Cardiovascular risk factors often coexist among older people. Our study suggests that an aggregation of multiple cardiovascular risk factors was strongly associated with both PAD and CAS. The magnitude of association is similar to the reports from another study [36]. This study suggests that older people with multiple cardiovascular risk factors may represent a subgroup at increased risk for PAD and CAS. Notably, we found that the cardiovascular risk factor profiles for PAD and CAS (e.g., increased cIMT) were slightly different. Hypertension and an increased LDL-C/HDL-C ratio were associated with both PAD and an increased cIMT. In addition, diabetes was independently associated with PAD but not with CAS, whereas smoking was associated with CAS but not with PAD. This implies that the etiology and pathophysiology of PAD and CAS may differ. Given that PAD and CAS are strongly associated with subsequent cardiovascular events [6], [14], intervention programs that target multiple modifiable cardiovascular risk factors such as smoking, hypertension, diabetes, and dyslipidemia among middle-aged and older people are likely to be effective in reducing the risk of cardiovascular disease later in life. Specifically, implementation of lifestyle interventions (e.g., weight reduction and physical activity) is desirable for cardiovascular disease prevention, whereas clinically pharmacological approaches are for treating multiple metabolic risk factors, including hyperglycemia, hypertension, and dyslipidemia [37], [38]. This is particularly relevant for China, considering the huge impact being faced from demographic transitions.

Strengths of this study include the community-based design, comprehensive assessment of extensive cardiovascular risk factors, and the use of standard approaches for defining PAD and assessing CAS. However, this study also has limitations. Primarily, the cross-sectional association of cardiovascular risk factors with PAD and CAS may be subject to selective survival bias, which may lead to the underestimation of the true association. Furthermore, we had limited power to estimate the associations of PAD with cardiovascular risk factors owing to the relatively too few subjects with PAD.

In conclusion, this population-based study suggests that among Chinese older people living in a rural community, PAD, carotid stenosis, and an increased cIMT are relatively uncommon. The likelihood of having PAD and CAS increases with the increasing number of certain cardiovascular risk factors. The cardiovascular risk factor profile for PAD is characterized by hypertension, diabetes, and an increased LDL-C/HDL-C ratio, whereas the profile for CAS is slightly different in which smoking, but not diabetes, is associated with CAS. This study shows that testing of intervention programs against atherosclerotic disorders by targeting those identified risk factors (e.g., smoking, hyperglycemia, hypertension, and dyslipidemia) is warranted.
